# An international comparative study of active living environments and hospitalization for Wales and Canada

**DOI:** 10.1016/j.ssmph.2022.101048

**Published:** 2022-02-25

**Authors:** Sarah M. Mah, Kaberi Dasgupta, Ashley Akbari, Nancy A. Ross, Richard Fry

**Affiliations:** a Department of Geography, McGill University, 705-805 Sherbrooke Street West, Montreal, Quebec, H3A 0B9, Canada; b Dalla Lana School of Public Health, University of Toronto, Health Sciences Building 155 College Street, 6th Floor Toronto, ON M5T 3M7, Canada; c Divisions of Internal Medicine, Divisions of Clinical Epidemiology, Divisions of Endocrinology and Metabolism. McGill University Health Centre, 1001 Decarie Boulevard, D02.3312, Montreal, Quebec, H4A 3J1, Canada; d Population Data Science, Swansea University Medical School, Swansea, UK; e Department of Public Health Sciences, School of Medicine, Queen's University, Carruthers Hall, 62 Fifth Field Company Lane, Kingston, ON, K7L 3N6, Canada

**Keywords:** Population health, Linked Data, Built environment, Walkability, Canada, Wales UK, Hospitalization, Morbidity, Active Living Environments

## Abstract

***Rationale:*** Previous studies indicate active living environments (ALEs) are associated with higher physical activity levels across different geographic contexts, and could lead to reductions in hospital burden. Both Wales UK and Canada have advanced data infrastructure that allows record linkage between survey data and administrative health information.

**Objective:**

To assess the relationship between ALEs and hospitalization in Wales and Canada.

**Methods:**

We performed a population-based comparison using individual-level survey data from the Welsh Health Survey (N = 9968) linked to the Patient Episode Database for Wales, and the Canadian Community Health Survey (N = 40,335) linked to the Discharge Abstract Database. Using equivalent protocols and open-source data for street networks, destinations, and residential density, we derived 5-class measures of the ALE for Wales and Canada (classed 1 through 5, considered least favourable to most favourable for active living, respectively). We evaluated relationships of ALEs to health, behaviours and hospitalization using multivariate regression (reference group was the lowest ALE class 1, considered least favourable for active living).

**Results:**

For Canada, those living in the highest ALE class 5 had lower odds of all-cause hospitalization (OR 0.66, 95% CI 0.54 to 0.81; as compared to the lowest ALE class 1). In contrast, those living in the highest ALE class 5 in Wales had higher odds of all-cause hospitalization (OR 1.37, 95% CI 1.04 to 1.80). The relationship between ALEs and cardiometabolic hospitalization was inconclusive for Canada (OR 0.75, 95% CI 0.50 to 1.12), but we observed higher odds of cardiometabolic hospitalization for respondents living in higher ALE classes for Wales (OR 1.46, 95% CI 1.10 to 1.78; comparing ALE class 4 to ALE class 1).

**Conclusion:**

Canadian respondents living in high ALE neighbourhoods that are understood to be favourable for active living had lower odds of all-cause hospitalization, whereas Welsh respondents living in high ALEs that were deemed favourable for active living exhibited higher odds of all-cause hospitalization. Environments which promote physical activity in one geographic context may not do so in another. There remains a need to identify relevant context-specific factors that encourage active living.

## Highlights

• Active living environments may support physical activity and population health across geographic contexts.
• The UK and Canada share similarities in population health trends.
• Living in more favourable neighbourhoods was associated with lower hospitalization in Canada. The opposite was true for Wales.
• Environmental characteristics which promote population health in one place may not do so in another.

## 1 Introduction

Rising obesity and chronic disease rates are among the most pervasive population health patterns emerging worldwide (Abarca-Gómez et al., 2017; NCD Risk Factor Collaboration, 2016b). The global prevalence of obesity increased from 3 to 10% in men and 6 to 15% in women since 1975 (NCD Risk Factor Collaboration, 2016a). Although obesity is driven, in part, by ageing populations, widespread physical inactivity is a major contributing factor (Guthold, Stevens, Riley, & Bull, 2018), especially in the context of urbanization and greater reliance on motorized vehicle transport (Kohl et al., 2012). There is increased interest in the impact of built environments on chronic diseases through the pathway of physical activity. Built environment factors that are understood to encourage active living in neighbourhoods often include a combination of greater street connectivity, more destinations such as parks, shops, and services, and high residential density; these can be calculated and measured using geographic information systems (GIS). Also referred to as neighbourhood *walkability*, favourable active living environments (ALEs) have been linked to higher step counts (Althoff et al., 2017; Hajna et al., 2015, Hajna et al., 2015) and favourable weight and cardiometabolic profiles in different parts of the world (Barbosa et al., 2019; Chandrabose et al., 2019). Area-level studies indicate that favourable neighbourhoods for active living could be linked with lower hospital burden and health care costs (Mazumdar et al., 2016; Yu et al., 2017). However, there are no individual-level studies that link ALEs and hospitalization to date. Moreover, much of the research on the health impacts of ALEs has been conducted in North America, which begs the question as to whether these associations are found in other contexts (Mackenbach et al., 2014).

International comparisons allow us to broaden the range and variability of ALEs under study, as well as examine how neighbourhood typologies operate in different regional contexts (Bilal, Auchincloss, & Diez-Roux, 2018). The International Physical Activity and the Environment Network (IPEN) study remains the key international cohort that demonstrated broadly positive associations between favourable neighbourhood attributes, physical activity, and lower levels of obesity (Sallis et al., 2020). However, such cohort studies tend to be limited in sample size and often do not have the capacity to assess ALEs against objectively measured health outcomes such as hospitalization and mortality. Few research initiatives can examine associations between environmental exposures and international health trends. This is due in part to the costs and challenges of obtaining analogous data on environmental exposures and downstream disease outcomes for multiple countries (Badland & Schofield, 2005). The growing adoption of record linkage approaches to address population health questions (Jutte, Roos, & Brownell, 2011) paired with the availability of locational information on many national health surveys is a promising and efficient means of achieving internationally comparable cohorts for studies of environments and health.

Wales and Canada have well-developed data linkage infrastructures capable of leveraging secondary administrative data to facilitate efficient, high-powered population-wide health research. Wales and Canada have distinct geographies, population sizes, histories, and cultural attitudes to physical activity and food (Croucher, Wallace, & Duffy, 2012). Nonetheless, in common with some other regions, Wales and Canada do have similarities. Both are wealthy nations with universal health care systems and mostly urban-dwelling populations (70% urban-dwelling for Wales, 80% for Canada). Wales and Canada have anticipated the need to invest in and adapt to their ageing populations (National Assembly for Wales Finance Committee, 2018), of which nearly a quarter will be 65 years or older by 2030 (Baxter & Boyce, 2011; Government of Canada, 2014). Obesity rates are comparable at 23% for the Welsh population age 16 years and older (Public Health Wales Observatory, 2019) and 27% for those age 18 years and older in Canada (Statistics Canada, 2019). Diabetes prevalence rates are identical at 7.3% for both Wales and Canada (Statistics Canada, 2018; StatsWales, 2016). In 2016/2017, most of the working population were car commuters in Wales (81%) and in Canada (74%) (Department for Transport, 2016; Yaropud et al., 2019). The primary objective of our study was to assess the associations between ALEs and hospital burden in Wales and in Canada, and evaluate whether the relationships were similar.

## 2 Methods

First, we described sociodemographic characteristics of each study sample, neighbourhood measures of the built environment for each country using a standardized ALE methodology, and hospitalization rates for each sample. We then conducted a cross-sectional assessment of relationships between ALEs and physical activity, obesity, and type 2 diabetes at baseline. Finally, we examined associations between ALEs and all-cause hospitalization as well as hospitalization for cardiometabolic disease. We hypothesized that those living in more favourable ALEs would exhibit lower odds of hospitalization for both countries.

### 2.1 Data sources

Two parallel analyses were conducted using population-based health surveys, administrative health data, and comparably derived ALE measures from Wales and Canada (Table 1). Each analysis was conducted within Trusted Research Environments (TREs) for linked microdata; the Secure Anonymised Information Linkage (SAIL) Databank for Wales and the Canadian Research Data Centres Network (CRDCN) for Canada. The Welsh Health Survey (WHS) and Canadian Community Health Survey (CCHS) are annual national cross-sectional surveys conducted by the National Centre for Social Research (commissioned by the Welsh Government) and Statistics Canada, respectively. Both surveys aim to provide population-wide estimates of health status as well as health-related behaviours and potential health determinants. As such, both collect similar information on socio-demographic factors, self-reported health and health behaviours. Data from WHS participants were linked with hospitalization records in the Patient Episode Database for Wales (PEDW), a register of all inpatient and day-case activity in National Health Service (NHS) Wales hospitals. CCHS participant data were linked with hospital records in the Discharge Abstract Database (DAD), a census of all acute inpatient hospitalizations to which all provinces submit data, except for the province of Quebec. Both hospitalization data sources contain information on date of admission, date of discharge, and diagnostic codes based on the 10th revision of the International Statistical Classification of Diseases and Related Health Problems (ICD-10).

**Table 1:** Linked data sources for Wales and Canada.

	WALES	CANADA
Linked data environment	SAIL Databank	CRDCN
Health survey	WHS	CCHS
Years available	2013–2014	2000–2011
Years used	2013–2014	2007
Annual target sample	10,000	65,000
Hospitalization	PEDW	DAD
Years available	2000–2017	1999–2012
Years used	2013–2017	2007–2011
Active Living Environments	Wales Active Living Environment Database (Wal-ALE)	Canadian Active Living Environment Database (Can-ALE)

### 2.2 Study population

We selected survey cycles and hospitalization fiscal years from each country to achieve, to the extent possible, comparable: 1) follow-up time for hospitalization, 2) sample size, and 3) time-period of the surveys. The years of the WHS available (2013, 2014) were more recent than those of the CCHS (2000–2011), and allowed for 3–4 years of hospitalization follow-up time to 2017 in the PEDW. Therefore, we selected CCHS 2007 as being the most recent cycle of the Canadian survey data that would render a similar hospitalization follow-up time of 3–4 years in the DAD after survey response. We also restricted the target age range of the CCHS (which surveys persons over the age of 12) to respondents aged 16 years and older to match that of the WHS.

### 2.3 Measure for the active living environment (ALE)

Each respondent's neighbourhood was linked with the Canadian Active Living Environments (Can-ALE) Database or the Welsh Active Living Environments (Wal-ALE) Database. Can-ALE is a nationally derived set of built environment measures for active living friendliness in Canada. The methodology has been described in detail (Herrmann et al., 2019) but briefly, neighbourhoods were delineated using 1000-meter Euclidean buffers around the centroids of dissemination areas (DA), which represent 400–700 persons and is the smallest geographic unit for which Canadian census data are released. Data on the road network and footpaths were downloaded from OpenStreetMap (OpenStreetMap Contributors, 2017) and used to calculate the density of ≥3-way intersections within each buffer to capture how well connected a neighbourhood is. The number of points of interest within each buffer was also calculated using Open Street Map data. Dwelling densities were retrieved from the 2016 Canadian census. Raw scores for the three components were clustered into five categories using k-medians, representing environments that are very active living-unfriendly (lowest ALE class 1) to those that are very active living-friendly (highest ALE class 5). Canadian survey respondents were then linked with their corresponding neighbourhoods by overlaying interpolated postal code coordinates (Wilkins & Peters, 2012) with 2016 DAs in a Geographic Information System (ArcGIS 10.5, ESRI).

Using the identical protocol for Can-ALE, the Welsh Active Living Environments (Wal-ALE) Database (Fry, Akbari, Mah, & Ross, 2018) was derived for Output Areas (OAs) corresponding to an average population of 300 people (with approximately 125 households) and is roughly comparable to the Canadian DA. Intersection density and points of interest components were derived using OpenStreetMap data, and dwelling density was calculated using data from the Office for National Statistics Usual Residents data. Source code and documentation for Wal-ALE are freely available at http://richfry.github.io/walkability/. Geospatial metrics were aggregated to Lower Layer Super Output Areas (LSOAs) for linking with data in the SAIL databank.

### 2.4 Outcomes

The primary outcome of interest was acute inpatient hospitalization. *Person-spells* for Wales and *episodes of care* for Canada were the units of analysis judged to be most relevant and comparable, and both units consider contiguous records for inpatient care as a single event. Records in the PEDW are organized into person-spells, while DAD records occurring within 24 hours of each other were considered part of the same episode. Day procedures from both Welsh and Canadian datasets were removed. Day case hospitalizations identified in the PEDW were identified as those with same-day start and end dates for a single person-spell. Day procedures in the DAD were removed from the analysis using the Facility Type Code variable, which are marked ‘A’ if the record is a day procedure. All-cause hospitalizations included admissions for any cause except for pregnancy or childbirth. We also tracked hospitalizations relating to broad classes of conditions where the following ICD-10 codes were present in the primary and/or secondary diagnostic positions: circulatory diseases (I00–I99), endocrine, nutritional and metabolic diseases (E00-E88), respiratory diseases (J00-J98) and cancer (C00-D48). Last, we tracked cardiometabolic hospitalizations, which we considered to be most plausibly related to physical inactivity using the following ICD-10 codes: I10-15 (hypertension), I20-25 (ischaemic heart disease), I61-69 (cerebrovascular disease), I50 (heart failure), and E11-E14 (diabetes, excluding type 1 diabetes).

We assessed physical inactivity, obesity, and type 2 diabetes as intermediate outcomes of interest. We defined “no exercise/leisure-time physical activity” as those who reported performing no exercise in the last week for Welsh survey respondents, and for Canadian respondents, no leisure-time physical activity in the last three months. For the CCHS, we also assessed walking for leisure and walking to work or school in supplementary analyses. Obesity was classified as body mass index exceeding 30 kg/m^2^, which was available as derived variables of self-reported height and weight in both the WHS and the CCHS.

### 2.5 Covariates and potential confounders

We considered age, sex, educational attainment, and smoking status (never, former, current) as potential confounders of the relationship between the ALEs and hospital burden. Age, sex, and smoking status were comparably coded for the two datasets. However, educational systems in Wales and Canada differ substantially. Therefore, variables for educational attainment in the WHS were very different from those in the CCHS. To harmonize these education variables for this analysis, we matched each educational level reported in the WHS or CCHS based on similarity in qualification and frequencies of people holding those qualifications between each country. We then regrouped these variables into three categories for each dataset. For Canada, these categories were defined as those with a postgraduate degree or diploma (“high”), those with high school graduation (“intermediate”), and those without high school graduation (“low”). For Wales, “high” educational attainment comprised of those who reported having a degree, professional qualification, foreign qualification, or vocational qualification of level 4 or higher, based on the Regulated Qualifications Framework (RQF), framework for Higher Education Qualifications (FHEQ), National Vocational Qualifications system, or other vocational/work-related qualification. We classified those who reported no qualifications as “low” educational attainment, and all remaining respondents as “intermediate.”

### 2.6 Statistical analysis

We generated baseline descriptive statistics of the cohorts and ALEs. We quantified hospital utilization over the follow-up period. Specifically, we calculated the proportion of people ever hospitalized after survey response for any cause and for selected causes (circulatory disease; endocrine, nutritional and metabolic diseases; respiratory diseases; cancers; cardiometabolic diseases), crude hospitalization rates per 100 person-years among those hospitalized (calculated by dividing aggregate hospitalization counts by the total time under observation of individuals, multiplied by 100), and average length of stay among those hospitalized.

We used logistic regression to assess the relationship between living in higher ALEs (with the lowest ALE class 1 as the reference group) and odds of reporting no leisure-time physical activity/exercise, obesity, and having type 2 diabetes at survey response. We then modelled the relationship between ALEs and odds of hospitalization for all causes and for cardiometabolic diseases over the follow-up period. We estimated predicted probabilities of each outcome for Wal-ALE and Can-ALE class 1 through 5 using the -margins- command, and calculated confidence intervals using the delta method. Models were run separately for Welsh and Canadian cohorts. All models were adjusted for sex, age, smoking status, and educational attainment, and included a quadratic term for age. We also used robust estimators of variance as well as an offset term to account for different follow-up times. All analyses were carried out using Stata version 15 (StataCorp, College Station, Texas 77845 USA). Note that in accordance with confidentiality protocols, all frequencies are reported using a rounding base of five for the Canadian sample, and therefore may not sum to rounded totals.

### 2.7 Ethics

This joint project was approved by: 1) the Information Governance Review Panel (IGRP, SAIL Agreement #0715), which reviews all proposals submitted to the SAIL Databank to ensure that they are appropriate and in the public interest, as well as 2) Statistics Canada (project number 16-HAD-MCG-4802, *Relationships between walkability, health behaviours and chronic disease outcomes: Validation of the CCHS walkability measure)*, which has in place a detailed protocol for the protection of respondent confidentiality that supersede the authority of Research Ethics Boards at Canadian universities (see https://www.statcan.gc.ca/en/microdata/data-centres/faq/mitigation).

## 3 Results

### 3.1 Population characteristics

The Welsh cohort was slightly older with lower educational attainment, less smoking, less obesity, and higher prevalence of diabetes, compared with the Canadian cohort (Table 2).

**Table 2:** Characteristics of study populations for Wales and Canada.

	No. (%)	
	Wales a (N = 9968)	Canada b (N = 40,335)
Age, mean (SD)	52.6 (18.5)	49.2 (18.8)
Age category (%)		
Younger 16-44	3367 (33.8)	17,105 (42.4)
Middle 45-64	3521 (35.3)	13,815 (34.3)
Older 65+	3080 (30.9)	9415 (23.3)
Female	5232 (52.5)	21,765 (54.0)
Educational attainment		
Low	1841 (18.5)	8645 (21.4)
Moderate	3310 (33.2)	10,205 (25.3)
High	4817 (48.3)	21,485 (53.3)
Smoking (%)		
Never	4880 (50.0)	13,520 (33.5)
Former	3208 (32.2)	17,285 (42.9)
Current	1880 (18.9)	9530 (23.6)
BMI (SD)	26.8 (5.4)	27.5 (5.6)
Obese (%)	2358 (23.7)	10,880 (27.0)
No exercise/leisure-time physical activity (%) c	1132 (11.4)	4115 (10.2)
Type 2 diabetes (%)	805 (8.1)	2960 (7.3)

BMI, Body Mass Index; SD, standard deviation.

a Data source: WHS.

b Data source: CCHS.

c For Wales, respondents who had not performed any exercise in the last 7-days before survey response. For Canada, respondents who had not performed any leisure-time physical activity in the last 3-months before survey response.

### 3.2 Neighbourhood characteristics

More Canadian neighbourhoods than Welsh neighbourhoods were classified as ALE class 1, neighbourhoods considered the least favourable for active living (Wales 36.9%; Canada 25.5%) (Table 3). A greater proportion of Canadian survey respondents lived in these neighbourhoods than Welsh respondents (Wales 28.5%; Canada 48.8%). A small proportion of respondents lived in neighbourhoods classified as ALE class 5, neighbourhoods considered most favourable for active living (Wales 3.6%; Canada 1.9%).

**Table 3:** Active living environments in Wales (Wal-ALE) and Canada (Can-ALE).

	
	ALE 1 a	ALE 2	ALE 3	ALE 4	ALE 5
Neighbourhoods					
Wal-ALE (LSOAs, %) b	484 (25.5)	517 (27.3)	510 (26.9)	279 (14.7)	106 (5.6)
Can-ALE (DAs, %) b	20,722 (36.9)	15,327 (27.3)	13,077 (23.3)	4541 (8.1)	2422 (4.3)
Respondents					
WHS (%) c	2838 (28.5)	2932 (29.4)	2700 (27.1)	1138 (11.4)	360 (3.6)
CCHS (%) c	19,670 (48.8)	11,045 (27.4)	7095 (17.6)	1750 (4.3)	780 (1.9)

ALE, active living environment; Wal-ALE, Welsh Active Living Environments; LSOA, lower-layer super output area; Can-ALE, Canadian Active Living Environments; DA, dissemination area; WHS, Welsh Health Survey; CCHS, Canadian Community Health Survey.

a Frequencies of ALE classes 1 (least favourable) through 5 (most favourable).

b The number and proportion of geographic units assigned to ALE classes.

c The number and proportion of survey respondents assigned to ALE classes.

**Fig. 1:**
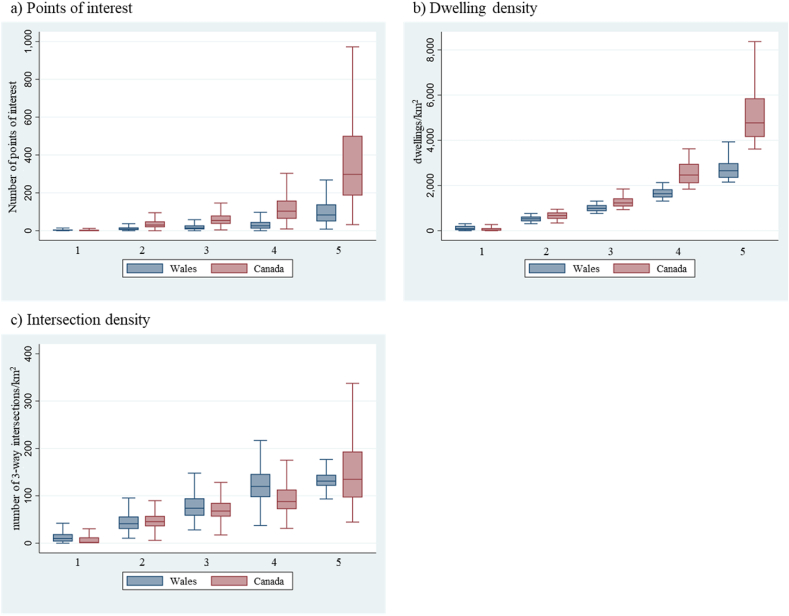
Boxplots of averages and distributions for points of interest (a), dwelling density (b), and intersection density (c) for Welsh LSOAs compared with Canadian DAs across ALE classes 1 (considered least favourable for active living) through 5 (considered most favourable for active living).

We observed more points of interest, greater dwelling density, and greater intersection density for higher ALE classes in both Canada and Wales (Fig. 1). The average number of points of interest and dwelling density were consistently higher across Can-ALE classes relative to Wal-ALE classes and were especially high in Can-ALE class 5 relative to Wal-ALE class 5. In contrast, average intersection density by Wal-ALE class appeared roughly equal to or higher than equivalent Can-ALE classes. Increases in intersection density with higher ALE also appeared to diminish after Can-ALE and Wal-ALE class 3.

We observed that ALE classes exhibited socioeconomic patterning by ALE class in Canada, but not in Wales (Table 4). Canadian respondents living in the highest ALEs had the highest education levels (high educational attainment: Can-ALE class 5–69.9%; Can-ALE class 1–50.3%). Such a pattern was not apparent for Wales. The lowest ALE class had the smallest proportion of current smokers in Wales (Wal-ALE class 1–14.7%) and the largest in Canada (Can-ALE class 1–25%). Mean age declined with increasing ALE class for both Wales and Canada (see Table 5).

**Table 4:** Demographics by ALE class.

	Wales (N = 9968) a					Canada (N = 40,335) b				
	ALE 1	ALE 2	ALE 3	ALE 4	ALE 5	ALE 1	ALE 2	ALE 3	ALE 4	ALE 5
N (%)	2838 (28.5)	2932 (29.4)	2700 (27.1)	1138 (11.4)	360 (3.6)	19,670 (48.8)	11,045 (27.4)	7095 (17.6)	1750 (4.3)	780 (1.9)
Age, mean (SD)	55.4 (17.8)	52.1 (18.7)	51.9 (18.5)	50.9 (18.7)	46.3 (18.8)	50.1 (18.3)	48.7 (19.1)	48.1 (19.3)	47.6 (19.0)	47.1 (18.3)
Female %	51.1	53.3	53.3	52.2	51.7	53.8	54.4	54.2	52.8	52.7
Educational attainment %										
Low	15.5	19.4	20.2	20.0	16.7	25.0	18.4	19.1	14.9	9.6
Moderate	30.5	34.3	34.3	35.8	29.4	24.7	26.1	26.1	26.2	20.4
High	54.0	46.3	45.5	44.3	53.9	50.3	55.5	54.9	58.9	69.9
Smoking %										
Never	50.7	48.6	48.4	46.1	50.8	30.6	34.3	38.3	40.3	37.9
Former	34.6	31.6	30.9	32.0	28.3	44.4	43.7	38.6	38.7	39.7
Current	14.7	19.8	20.7	21.9	20.8	25.0	22.0	23.1	21.0	22.4
Obese %	20.7	25.1	24.9	25.5	20.3	30.1	26.2	23.4	18.2	11.7
No exercise/leisure-time physical activity (%) c	9.1	11.5	12.7	12.5	15.0	10.8	9.2	9.8	11.7	10.8
Type 2 diabetes %	7.3	8.9	8.5	7.2	7.2	7.8	6.9	7.0	7.2	5.4

ALE, active living environment; SD, standard deviation.

a Data sources: WHS, Wal-ALE.

b Data sources: CCHS, Can-ALE.

c For Wales, respondents who had not performed any exercise in the last 7 days before survey response. For Canada, respondents who had not performed any leisure-time physical activity in the last 3 months before survey response.

**Table 5:** Hospitalization trends in Welsh and Canadian samples.

	Wales (N = 9968) a	Canada (N = 40,335) b
Total time under observation, years	38,561	179,203
Average time under observation, years (SD)	3.9 (0.7)	4.4 (0.6)
Hospitalized (%) d		
All-cause	2334 (23.4)	10,235 (25.4)
Circulatory diseases	666 (6.7)	2815 (7.0)
ENM diseases	229 (2.3)	1035 (2.6)
Respiratory diseases	444 (4.5)	1500 (3.7)
Cancers	428 (4.3)	1655 (4.1)
Cardiometabolic diseases	559 (5.6)	2265 (5.6)
Hospitalization rate per 100 PY d		
All-cause	11.8	11.7
Circulatory diseases	2.3	2.3
ENM diseases	0.8	0.8
Respiratory diseases	1.7	1.3
Cancers	2.0	1.2
Cardiometabolic diseases	1.8	1.9
Average length of stay, days (SD)		
All-cause	10.0 (24.2)	9.3 (20.1)
Circulatory diseases	9.7 (21.4)	10.0 (17.0)
ENM diseases	7.6 (13.4)	10.2 (18.5)
Respiratory diseases	9.9 (15.8)	10.4 (20.2)
Cancers	9.4 (14.8)	9.9 (16.7)
Cardiometabolic diseases	8.6 (18.0)	11.0 (17.8)

PY, person-years; SD, standard deviation, ENM; Endocrine, nutritional and metabolic diseases.

^c^ Number of hospitalized individuals, (%) is the proportion of people hospitalized.

a Data source: WHS, PEDW.

b Data source: CCHS, DAD.

d Aggregate number of hospitalizations per 100 person-years calculated by dividing the total number of hospitalizations by total person time.

The proportion of respondents with obesity decreased incrementally with higher ALE classes for Canada, while Welsh respondents living in intermediate ALE classes exhibited the highest obesity. The proportion of those reporting no exercise was highest in the highest Wal-ALE. No clear pattern was seen for people reporting no leisure-time physical activity across ALE classes in Canada. However, walking measures that were only available for the Canadian cohort showed that more people walked in incrementally higher ALE classes for Canada (Supplementary material, Table S1). Type 2 diabetes was relatively stable across ALE classes, aside from the smaller proportion of those who reported having type 2 diabetes in the highest ALE class relative to those living in the lowest ALE class (Can-ALE class 5–5.4%; Can-ALE class 1–7.8%).

### 3.3 Hospitalization characteristics

We observed similar hospitalization rates across categories for Wales and Canada, including all-cause (11.8 and 11.7 hospitalizations per 100 person-years), circulatory (both 2.3 hospitalizations per 100 person-years), endocrine, nutritional, and metabolic diseases (both 0.8 hospitalizations per 100 person-years), and other causes (in hospitalizations per 100 person-years: 1.7 and 1.3 for respiratory diseases; 2.0 and 1.2 for cancers; 1.8 and 1.9 for cardiometabolic diseases, for Wales and Canada respectively). Cause-specific length of stay was consistently shorter in Wales than in Canada.

### 3.4 Associations of ALEs, health, and hospitalization

Higher ALEs were associated with lower odds of obesity in Canada, higher odds of obesity in Wales, and higher odds of reporting no physical activity in both Wales and Canada (Table 6). Models showed small differences across ALE classes for odds of type 2 diabetes for Canada, but indicated heightened odds of type 2 diabetes for those living in higher ALEs for Wales.

**Table 6:** ALEs and baseline odds of no exercise/leisure-time physical activity, obesity, and type 2 diabetes.

	Wales (N = 9968) b		Canada (N = 40,335) c	
	OR (95% CI) a	Predicted d	OR (95% CI) a	Predicted d
No exercise/leisure-time physical activity (yes/no) d				
ALE 1	1.00	0.09 (0.08, 0.10)	1.00	0.10 (0.10, 0.11)
ALE 2	1.25 (1.04, 1.50) *	0.11 (0.10, 0.12)	0.92 (0.84, 0.99) *	0.10 (0.09, 0.10)
ALE 3	1.40 (1.16, 1.67) *	0.12 (0.11, 0.14)	0.97 (0.88, 1.06)	0.10 (0.09, 0.11)
ALE 4	1.39 (1.10, 1.74) *	0.12 (0.10, 0.14)	1.27 (1.08, 1.49) *	0.13 (0.11, 0.14)
ALE 5	2.01 (1.43, 2.81) *	0.17 (0.13, 0.20)	1.26 (0.99, 1.60)	0.13 (0.10, 0.15)
Obesity (yes/no)				
ALE 1	1.00	0.21 (0.19, 0.22)	1.00	0.29 (0.29, 0.30)
ALE 2	1.31 (1.15, 1.48) *	0.25 (0.24, 0.27)	0.87 (0.82, 0.92) *	0.27 (0.26, 0.27)
ALE 3	1.29 (1.13, 1.47) *	0.25 (0.23, 0.26)	0.76 (0.71, 0.81) *	0.24 (0.23, 0.25)
ALE 4	1.35 (1.15, 1.60) *	0.26 (0.23, 0.28)	0.55 (0.49, 0.63) *	0.19 (0.17, 0.21)
ALE 5	1.06 (0.80, 1.40)	0.21 (0.17, 0.26)	0.33 (0.26, 0.41) *	0.12 (0.10, 0.15)
Type 2 diabetes (yes/no)				
ALE 1	1.00	0.07 (0.06, 0.07)	1.00	0.07 (0.07, 0.08)
ALE 2	1.44 (1.18,1.76) *	0.09 (0.08, 0.10)	0.97 (0.89, 1.07)	0.07 (0.07, 0.08)
ALE 3	1.40 (1.14,1.72) *	0.09 (0.08, 0.10)	1.02 (0.91, 1.13)	0.07 (0.07, 0.08)
ALE 4	1.20 (0.91,1.58)	0.08 (0.06, 0.09)	1.13 (0.92, 1.38)	0.08 (0.07, 0.09)
ALE 5	1.56 (0.99,2.44)	0.10 (0.06, 0.13)	0.86 (0.62, 1.20)	0.06 (0.05, 0.08)

OR, Odds ratios; CI, confidence interval; ALE, active living environment.

^e^ Marginal predicted probability of being sedentary, obese, or having type 2 diabetes at baseline. Confidence intervals calculated using the delta method.

^f^ ALE classes 1 (least favourable) to 5 (most favourable). Wal-ALE was used for models with the Welsh cohort, and Can-ALE was used for models with the Canadian cohort.

a All models for both Welsh and Canadian cohorts adjusted for age, sex, educational attainment, and smoking status.

b Data source: WHS, PEDW.

c Data source: CCHS, DAD.

d For Wales, respondents who had not performed any exercise in the last 7 days before survey response. For Canada, respondents who had not performed any leisure-time physical activity in the last 3 months before survey response.

Overall, higher Can-ALEs were associated with lower odds of all-cause hospitalization for Canadian respondents (OR 0.66, 95% CI 0.54 to 0.81, comparing Can-ALE class 5 to class 1), while higher Wal-ALEs were associated with greater odds of all-cause hospitalization for Welsh respondents (OR 1.37, 95% CI 1.04 to 1.80, comparing Wal-ALE class 5 to class 1) (Table 7). The relationship between ALEs and cardiometabolic hospitalization was inconclusive for Canada (OR 0.75, 95% CI 0.50 to 1.12, comparing Can-ALE class 5 to class 1). Higher Wal-ALEs were associated with greater odds of cardiometabolic hospitalization for Welsh respondents (OR 1.46, 95% CI 1.10 to 1.78, comparing Wal-ALE class 4 to class 1).

**Table 7:** Adjusted models of the relationship between ALEs and odds of hospitalization.

	Wales b		Canada c	
	OR (95% CI) a	Predicted d	OR (95% CI) a	Predicted^d^
*All-cause hospitalization*				
ALE 1 e	1.00	0.22 (0.21, 0.23)	1.00	0.27 (0.26, 0.27)
ALE 2	1.09 (0.96, 1.25)	0.23 (0.22, 0.25)	0.90 (0.85, 0.95) *	0.25 (0.24, 0.26)
ALE 3	1.16 (1.01, 1.33) *	0.24 (0.23, 0.26)	0.81 (0.75, 0.87) *	0.23 (0.22, 0.24)
ALE 4	1.18 (0.99, 1.41)	0.25 (0.22, 0.27)	0.78 (0.68, 0.88) *	0.23 (0.21, 0.25)
ALE 5	1.37 (1.04, 1.80) *	0.27 (0.23, 0.32)	0.66 (0.54, 0.81) *	0.20 (0.18, 0.23)
*Cardiometabolic hospitalization*				
ALE 1	1.00	0.05 (0.04, 0.06)	1.00	0.06 (0.05, 0.06)
ALE 2	1.27 (1.14, 1.63) *	0.06 (0.05, 0.07)	1.03 (0.93, 1.15)	0.06 (0.05, 0.06)
ALE 3	1.19 (1.04, 1.51)	0.06 (0.05, 0.06)	0.88 (0.77, 1.01)	0.05 (0.05, 0.06)
ALE 4	1.46 (1.10, 1.78) *	0.07 (0.05, 0.08)	0.83 (0.65, 1.07)	0.05 (0.04, 0.06)
ALE 5	1.51 (1.04, 2.36)	0.07 (0.04, 0.10)	0.75 (0.50, 1.12)	0.04 (0.03, 0.06)

OR, Odds ratios; CI, confidence interval; ALE, active living environment.

a All models for both Welsh and Canadian cohorts adjusted for age, sex, educational attainment, and smoking status.

b Data source: WHS, PEDW.

c Data source: CCHS, DAD.

d Marginal predicted probability of being hospitalized during the study period. Confidence intervals calculated using the delta method.

e ALE classes 1 (least favourable) to 5 (most favourable). Wal-ALE was used for models with the Welsh cohort and Can-ALE was used for models with the Canadian cohort.

## 4 Discussion

### 4.1 Main findings

Our goal was to explore associations between comparably derived ALE measures and hospitalization in two different countries. Based on previous international studies signalling widespread benefit of walkable environments for physical activity, we hypothesized that higher ALEs, understood to be more favourable for active living, would be associated with lower hospitalization in both countries. Socioeconomic status appeared stratified by ALE class in Canada but not Wales. Those living in higher ALE classes in Canada were more educated. Current smokers were fewest in the lowest ALE class for Wales, while the opposite was true for Canada; the greatest proportion of current smokers were found in the lowest ALE class. Cross-sectional analyses indicated that living in higher ALEs was associated with lower odds of obesity at baseline for the Canadian sample, but not for the Welsh sample. Finally, we demonstrated that respondents living in higher ALE classes in Canada had lower odds of all-cause hospitalization. Unexpectedly, the opposite was true for Wales; those living in the highest ALE classes exhibited the greatest odds of all-cause hospitalization.

Canadian respondents living in higher ALEs had lower odds of obesity at baseline and lower odds of hospitalization over the follow-up period. In contrast, Welsh participants living in higher ALEs had greater odds of obesity and hospitalization over the follow-up period. In neither region did the presumed active living friendliness of neighbourhoods actually enhance physical activity – as measured by the physical activity variables we identified as being most comparable between the two cohorts. While living in neighbourhoods defined as more favourable for active living was associated with lower obesity and hospitalization rates in Canada, the converse was true in Wales. This suggests that the ALE concept cannot simply be transplanted from one region to another without careful examination.

Our findings for Canada echo the results of two previous studies conducted in a single administrative region of Australia. Neighbourhoods with higher Walk Scores® were associated with reduced hospitalization for acute myocardial infarction (Mazumdar et al., 2016), as well as lower admissions and hospital costs per person (Yu et al., 2017). In this study, we observed conclusively lower odds of all-cause hospitalization for those living in higher ALEs in Canada. Cardiometabolic hospitalizations also appeared marginally lower for those living in higher Can-ALEs based on point estimates, but these estimates were inconclusive and underpowered. We also found a conclusive relationship between higher ALE and lower obesity; an inverse pattern that has been well-documented for Canada (Colley, Christidis, Michaud, Tjepkema, & Ross, 2019; Wasfi, Dasgupta, Orpana, & Ross, 2016). Although we found that residents of higher ALEs were the most likely to report doing no leisure-time physical activity, this result, as well as our supplementary analyses of walking in this study, are consistent with previous Canadian evidence that suggests ALEs are associated with walking (Hajna, Ross, Joseph, Harper, & Dasgupta, 2015) but not overall leisure-time physical activity (Thielman, Rosella, Copes, Lebenbaum, & Manson, 2015). Leisure-time physical activity can encompass a variety of different activities and therefore, may be less sensitive to built environments.

Unlike Canada, ALEs for Wales operated very differently with respect to hospitalization. Those living in higher Wal-ALEs exhibited the greatest odds of all-cause hospitalization and hospitalization for cardiometabolic disease. Respondents living in the highest ALEs were also most likely to report no exercise, while those in intermediate ALE classes were more likely to report obesity as well as having type 2 diabetes.

There are several insights that may help us understand these results. Applying a methodology for measuring ALEs in Wales - one that was developed and validated for a different country, makes strong assumptions about how people interact with built environments. The contrasting relationships of ALEs and health outcomes in Wales compared with Canada indicates that the significance of ALEs to health and health-related behaviours is likely to differ. Furthermore, the ALE is fundamentally an urban concept that is based on street network, destinations and population density; characteristics that embody cities. As a result, the measure does not capture other important health-promoting (nor health-damaging) aspects of rural areas that might benefit physical activity. For example, the presence of green-blue space and access to natural environments have been linked with positive health outcomes (Frumkin et al., 2017; Markevych et al., 2017; Mizen et al., 2019) and are characteristic of many rural neighbourhoods in Wales. Such rural features may impart physical activity benefits that contribute to the lower obesity and hospitalization rates we observed for those living in Welsh neighbourhoods we measured as being “less favourable” for active living based on the connectivity, destinations and density alone.

We must also take stock of the differences in the historical and geographical contexts of these two countries. Unique developmental histories have shaped the urban fabric of Wales and Canada in ways that influence how people interact with their neighbourhoods. Presently, Wales has a population of 3.1 million covering an area roughly the size of the US state of New Jersey at 20,782 square kilometers. The enduring street network was designed for pedestrian foot traffic (Boerefijn, 2010). Much of the urban and peri-urban areas of southern Wales were marked by severe economic depression after the decline of coal, and later developed a reputation for being overcrowded, impoverished, and less desirable as places to live, compared with their more picturesque rural counterparts (Cherry & Rogers, 2003). Historic underinvestment in urban and peri-urban areas and the persistence of small rural towns have likely produced lower and more consistent population densities and points of interest across Wal-ALE classes. In contrast, the Canadian population is over ten-fold that of Wales at nearly 37.6 million and spans 9.985 million square kilometers. Most of Canada is subject to harsh weather conditions, and its cities arose in areas with good agricultural land that were also more favourably positioned for ease of trade close to the Canada-US border. The country has also recognized the potential drawbacks of car-oriented, low-density urban sprawl, and has made considerable efforts to make cities public transit-oriented. Canada's more recent history, the sheer size of the country as well as the heterogeneity across regions and provinces partly explains the substantial demarcation in densities and points of interest across Can-ALE classes compared with Wal-ALE classes. Such geospatial measures are unable to capture these historic and socio-cultural factors that have shaped the built environments of Wales and Canada in distinct ways and in turn, the ways and extent to which neighbourhoods influence health and health-related behaviours.

### 4.2 Strengths and limitations

There are several strengths to our study. We leveraged pre-existing health data and open-source environmental data to extract detailed information for two large sample sizes, and we were able to carry out analyses in parallel for two different countries. We used objectively assessed exposure variables (Can-ALE and Wal-ALE measures) as well as outcomes (routine impatient hospitalization records) that were comparably derived using similar data sources and calculation protocols – features that address the limitations of using subjective measures of health status and neighbourhood characteristics (i.e. measurement error, recall bias). We chose national health surveys that bear similar questionnaire items, and we further harmonized variables to strengthen the extent to which descriptive statistics as well as model-based associations could be compared across the two countries. Overall, this study demonstrates the value and efficiency that record linkage initiatives bring to future international comparative research, and underscores the importance of secondary data as well as comparably derived environmental measures across countries.

Our approach also has limitations. First, analyses with record-linked data are often conducted in TREs due to the sensitive nature of individual-level microdata. Such was the case for this project. Parallel analyses were carried out on distinct secure TREs on which the linked data are housed, which prohibited us from generating pooled estimates for the Canadian and Welsh respondents as a single sample. While this may present a challenge to future international studies, the focus of this paper was comparisons of within-country associations of ALE and hospitalization. Second, the UK and Canada have substantially different health care systems with their own administrative data collection, classification, and structuring practices. With respect to this study, Wales operates under a single devolved health care system of the NHS, while Canada's health care system is decentralized, separately administered by thirteen provinces and territories. These systemic differences were a likely driver of the small but present inconsistencies in hospitalization metrics between Wales and Canada – particularly that seen for average length of stay. For example, the PEDW contains all inpatient and day case activity undertaken in NHS Wales. In contrast, Canada's DAD covers only acute inpatient hospitalizations. We took steps to account for these variations, such as excluding likely day case activity in the PEDW, and we arrived at similar all-cause hospitalization rates for both countries. Although more work is needed to assess the compatibility of these hospitalization data, it is unlikely that these systemic differences are biased with respect to their association with ALEs. Third, residential self-selection remains a perennial challenge for studies of health and the built environment. Proxy measures for self-selection such as neighbourhood preference are increasingly being developed but were not collected in these surveys. We note, however, that many previous studies indicate neighbourhood environments remain important determinants of physical activity, even after accounting for residential self-selection (Lamb et al., 2020). Lastly, there were important differences between the variables of the WHS and CCHS that further limit comparability. These include sample size (the Welsh sample size was roughly a quarter of the Canadian sample), differences in average follow-up time (longer for Canadian respondents), and temporal differences in the survey years available for analysis (2013–2014 for the WHS, 2007 for the CCHS). The measures of physical activity were also different for each cohort. The survey items related to “exercise” in the WHS versus “leisure-time physical activity” in the CCHS are likely to test different concepts of physical activity (Caspersen, Powell, & Christenson, 1985). Furthermore, the two variables differed in recall time, which could impact whether the variables capture usual versus current behaviour (Sternfeld & Goldman-Rosas, 2012). The exercise variable in the WHS asked respondents to include all exercise over the last 7 days (more likely to capture current behaviour), while the leisure-time physical activity variable in the CCHS asked about frequency and duration of activities over the last 3 months (more likely to capture usual behaviour). Unlike the CCHS, specific physical activity variables such as walking were not collected for the WHS. Therefore, whether walking (or other specific forms of physical activity) might be impacted by ALEs remains unknown for Wales. Comparable survey and administrative data remain challenging to attain. There remains a need for international benchmarks for the collection of health and demographic data.

### 4.3 Wider implications

This comparative study of secondary linked administrative health data allowed us to gain insight, with considerable power and efficiency, into the potential for ALEs to support population health in places where less research has been conducted on built environments. Our study shows that environments which are presumed to promote physical activity in one place may not function in the same way in another place. Among the sociodemographic, health and behavioural variations across each country's ALE classes, it appears that the role of ALEs in shaping behaviours and health is evident for Canada but not for Wales. Although this study appears to contradict previous international studies demonstrating strong associations between walkable neighbourhoods and physical activity, it is important to bear in mind that previous studies were mostly conducted in select cities and urban contexts. It is also important to acknowledge that the ALE concept arose in response to the unique urban challenges of North America, and indeed may be of less relevance to other geographies (Mackenbach et al., 2014).

This work confronts the limitations of our current understanding of ALEs, the generalizability of associations with positive outcomes for health and health behaviours, and our current methods for measuring ALEs. It implies that designating a group of built environment features as determinants of active living could be misguided if these features are not actually correlated with active living in all jurisdictions. As it stands, the combination of street connectivity, destinations, and density does not capture what favours physical activity and related health outcomes in Wales. While there is great value in acknowledging the widespread benefit of ALEs across a range of different geographies, there remains a need to identify relevant context-specific factors that encourage active living, underlining the importance of context-specific policy approaches to urban planning and the built environment.

## Ethical statement

This joint project was approved by: 1) the Information Governance Review Panel (IGRP, SAIL Agreement #0715), which reviews all proposals submitted to the SAIL Databank to ensure that they are appropriate and in the public interest, as well as 2) Statistics Canada (project number 16-HAD-MCG-4802, *Relationships between walkability, health behaviours and chronic disease outcomes: Validation of the CCHS walkability measure)*, which has in place a detailed protocol for the protection of respondent confidentiality that supersede the authority of Research Ethics Boards at Canadian universities (see http://www.statcan.gc.ca/eng/rdc/mitigation).

## Author statement

**Sarah M Mah:** Conceptualization, Methodology, Formal Analysis, Investigation, Writing – Original draft preparation. **Richard Fry**: Conceptualization, Methodology, Validation, Formal Analysis, Resources, Writing- Review and Editing. **Ashley Akbari**: Conceptualization, Resources, Data Curation, Writing- Review and Editing. **Nancy A Ross***:* Conceptualization, Supervision, Writing - Review & Editing, Funding Acquisition. **Kaberi Dasgupta:** Conceptualization, Supervision, Writing - Review & Editing.

## References

[bib1] Abarca-Gómez L., Abdeen Z.A., Hamid Z.A., Abu-Rmeileh N.M., Acosta-Cazares B., Acuin C., Aguilar-Salinas C.A. (2017). Worldwide trends in body-mass index, underweight, overweight, and obesity from 1975 to 2016: A pooled analysis of 2416 population-based measurement studies in 128· 9 million children, adolescents, and adults. The Lancet.

[bib2] Althoff T., Sosic R., Hicks J.L., King A.C., Delp S.L., Leskovec J. (2017). Large-scale physical activity data reveal worldwide activity inequality. Nature.

[bib3] Badland H., Schofield G. (2005). Transport, urban design, and physical activity: An evidence-based update. Transportation Research Part D: Transport and Environment.

[bib4] Barbosa J., Guerra P.H., Santos C.D., Nunes A., Turrell G., Florindo A.A. (2019). Walkability, overweight, and obesity in adults: A systematic review of observational studies. International Journal of Environmental Research and Public Health.

[bib5] Baxter J., Boyce S., Roberts O. (2011). *Key Issues for the fourth Assembly*: Research service national Assembly for Wales.

[bib6] Bilal U., Auchincloss A.H., Diez-Roux A.V. (2018). Neighborhood environments and diabetes Risk and control. Current Diabetes Reports.

[bib7] Boerefijn W.N.A. (2010).

[bib8] Caspersen C.J., Powell K.E., Christenson G.M. (1985). Physical activity, exercise, and physical fitness: Definitions and distinctions for health-related research. Public health reports.

[bib9] Chandrabose M., Rachele J.N., Gunn L., Kavanagh A., Owen N., Turrell G., Sugiyama T. (2019). Built environment and cardio-metabolic health: Systematic review and meta-analysis of longitudinal studies. Obesity Reviews.

[bib10] Cherry G., Rogers A.W. (2003).

[bib11] Colley R.C., Christidis T., Michaud I., Tjepkema M., Ross N.A. (2019). An examination of the associations between walkable neighbourhoods and obesity and self-rated health in Canadians. Health Reports.

[bib12] Contributors O.S.M. (2017). https://www.OpenStreetMap.org.

[bib13] Croucher K., Wallace A., Duffy S. (2012).

[bib14] Department for Transport (2016). https://www.gov.uk/government/statistics/transport-statistics-great-britain-2016.

[bib15] Frumkin H., Bratman G.N., Breslow S.J., Cochran B., Kahn P.H., Lawler J.J., Wood S.A. (2017). Nature contact and human health: A research agenda. Environmental Health Perspectives.

[bib16] Fry R., Akbari A., Mah S., Ross N. (2018). Measuring active living environments: An international comparison between Canada and Wales. International Journal of Population Data Science.

[bib17] Government of Canada (2014). https://www.canada.ca/en/employment-social-development/programs/seniors-action-report.html.

[bib18] Guthold R., Stevens G.A., Riley L.M., Bull F.C. (2018). Worldwide trends in insufficient physical activity from 2001 to 2016: A pooled analysis of 358 population-based surveys with 1.9 million participants. Lancet Global Health.

[bib19] Hajna S., Ross N.A., Brazeau A.S., Belisle P., Joseph L., Dasgupta K. (2015). Associations between neighbourhood walkability and daily steps in adults: A systematic review and meta-analysis. BMC Public Health.

[bib20] Hajna S., Ross N.A., Joseph L., Harper S., Dasgupta K. (2015). Neighbourhood walkability, daily steps and utilitarian walking in Canadian adults. BMJ Open.

[bib21] Herrmann T., Gleckner W., Wasfi R.A., Thierry B., Kestens Y., Ross N.A. (2019). A pan-Canadian measure of active living environments using open data. Health Reports.

[bib22] Jutte D.P., Roos L.L., Brownell M.D. (2011). Administrative record linkage as a tool for public health research. Annual Review of Public Health.

[bib23] Kohl H.W., Craig C.L., Lambert E.V., Inoue S., Alkandari J.R., Leetongin G., Lancet Physical Activity Series Working, G (2012). The pandemic of physical inactivity: Global action for public health. The Lancet.

[bib24] Lamb K.E., Thornton L.E., King T.L., Ball K., White S.R., Bentley R., Daniel M. (2020). Methods for accounting for neighbourhood self-selection in physical activity and dietary behaviour research: A systematic review. International Journal of Behavioral Nutrition and Physical Activity.

[bib25] Mackenbach J.D., Rutter H., Compernolle S., Glonti K., Oppert J.M., Charreire H., Lakerveld J. (2014). Obesogenic environments: A systematic review of the association between the physical environment and adult weight status, the SPOTLIGHT project. BMC Public Health.

[bib26] Markevych I., Schoierer J., Hartig T., Chudnovsky A., Hystad P., Dzhambov A.M., Fuertes E. (2017). Exploring pathways linking greenspace to health: Theoretical and methodological guidance. Environmental Research.

[bib27] Mazumdar S., Learnihan V., Cochrane T., Phung H., O'Connor B., Davey R. (2016). Is Walk score associated with hospital admissions from chronic diseases? Evidence from a cross-sectional study in a high socioeconomic status Australian city-state. BMJ Open.

[bib28] Mizen A., Song J., Fry R., Akbari A., Berridge D., Parker S.C., Rodgers S.E. (2019). Longitudinal access and exposure to green-blue spaces and individual-level mental health and well-being: Protocol for a longitudinal, population-wide record-linked natural experiment. BMJ Open.

[bib29] National Assembly for Wales Finance Committee (2018). The cost of caring for an ageing population. https://senedd.wales/laid%20documents/cr-ld11773/cr-ld11773-e.pdf.

[bib30] NCD Risk Factor Collaboration (2016). Trends in adult body-mass index in 200 countries from 1975 to 2014: A pooled analysis of 1698 population-based measurement studies with 19·2 million participants. The Lancet.

[bib31] NCD Risk Factor Collaboration (2016). Worldwide trends in diabetes since 1980: A pooled analysis of 751 population-based studies with 4·4 million participants. The Lancet.

[bib32] Public Health Wales Observatory (2019). http://www.publichealthwalesobservatory.wales.nhs.uk/obesityinwales.

[bib33] Sallis J.F., Cerin E., Kerr J., Adams M.A., Sugiyama T., Christiansen L.B., Owen N. (2020). Built environment, physical activity, and obesity: Findings from the international physical activity and environment network (IPEN) adult study. Annual Review of Public Health.

[bib34] Statistics Canada (2018). https://www150.statcan.gc.ca/n1/pub/82-625-x/2018001/article/54982-eng.htm.

[bib35] Statistics Canada (2019). https://www150.statcan.gc.ca/n1/pub/82-625-x/2019001/article/00005-eng.pdf.

[bib36] StatsWales (2016). https://statswales.gov.wales/Catalogue/Health-and-Social-Care/Welsh-Health-Survey/illnesses-by-gender-year.

[bib37] Sternfeld B., Goldman-Rosas L. (2012). A systematic approach to selecting an appropriate measure of self-reported physical activity or sedentary behavior. Journal of Physical Activity and Health.

[bib38] Thielman J., Rosella L., Copes R., Lebenbaum M., Manson H. (2015). Neighborhood walkability: Differential associations with self-reported transport walking and leisure-time physical activity in Canadian towns and cities of all sizes. Preventive Medicine.

[bib39] Wasfi R.A., Dasgupta K., Orpana H., Ross N.A. (2016). Neighborhood walkability and body mass index trajectories: Longitudinal study of Canadians. American Journal of Public Health.

[bib40] Wilkins R., Peters P.A. (2012).

[bib41] Yaropud T., Gilmore J., LaRochelle-Côté S. (2019). https://www150.statcan.gc.ca/n1/pub/75-006-x/2019001/article/00002-eng.htm.

[bib42] Yu Y., Davey R., Cochrane T., Learnihan V., Hanigan I.C., Bagheri N. (2017). Neighborhood walkability and hospital treatment costs: A first assessment. Preventive Medicine.

