# Supplementary material for: An international comparative study of active living environments and hospitalization for Wales and Canada
Source: SSM Popul Health. 2022 Feb 25;18:101048. doi: 10.1016/j.ssmph.2022.101048 (PMC8965167; doi:10.1016/j.ssmph.2022.101048)
Supplement: Multimedia component 1 [file mmc1.docx]

**Supplementary Materials to:** An International Comparative Study of Active Living Environments and Hospitalization for Wales and Canada

| Table S1. Physical activity and walking by ALE class in Canada | | | | | |
| --- | --- | --- | --- | --- | --- |
|  | ALE 1 | ALE 2 | ALE 3 | ALE 4 | ALE 5 |
|  |  |  |  |  |  |
| No LTPA/exercise % ^a^ | 10.8 | 9.2 | 9.8 | 11.67 | 10.80 |
|  |  |  |  |  |  |
| No leisure walking % ^b^ | 29.7 | 26.4 | 26.9 | 28.2 | 26.1 |
|  |  |  |  |  |  |
| No walking to work/school % ^c^ | 89.6 | 87.4 | 84.3 | 78.6 | 65.0 |
|  |  |  |  |  |  |
| No walking % ^d^ | 27.1 | 23.4 | 23.2 | 23.4 | 16.9 |
|  |  |  |  |  |  |
| Note there are some non-responses to the walking variables. Sample size differences are summarized in the footnotes below.  ^a^ N = 40,355 (full sample).  ^b^ N = 40,045.  ^c^ N = 40,235.  ^d^ No leisure walking or walking to work/school. N = 40,040.  Data sources: CHHS, Can-ALE.  ALE, active living environment. | | | | | |
